# “It Takes Two to Tango”: Role of Neglected Macrophage Manipulators Coronin 1 and Protein Kinase G in Mycobacterial Pathogenesis

**DOI:** 10.3389/fcimb.2020.582563

**Published:** 2020-10-20

**Authors:** Saradindu Saha, Payel Das, Somdeb BoseDasgupta

**Affiliations:** Molecular Immunology and Cellular Microbiology Laboratory, Department of Biotechnology, Indian Institute of Technology Kharagpur, Kharagpur, India

**Keywords:** mycobacterium, macrophage, Coronin 1, PknG, phagosome arrest, host-directed therapy

## Abstract

Macrophages being the connecting link between innate and adaptive immune system plays a crucial role in microbial antigen presentation and orchestrates the subsequent clearance of microorganisms. Microbial invasion of macrophages trigger a plethora of signaling cascades, which interact among them to generate a dynamically altered hostile environment, that ultimately leads to disruption of microbial pathogenesis. Paradoxically, *Mycobacterium sp*. exploits macrophage proteins such as Coronin 1, Calcineurin, LRG47, SOCS1, CISH, Gbp5 etc. and secretes virulence proteins such as PknG, PtpA, SapM, Eis etc. to hijack these intra-macrophage, signaling cascades and thereby develop its own niche. Coronin 1, being a cortical protein is transiently recruited to all mycobacteria containing phagosomes, but only pathogenic mycobacteria can retain it on the phagosome, to hinder its maturation. Additionally, mycobacterial infection linked secretion of virulence factor Protein Kinase G through its phosphorylation, manipulates several macrophage signaling pathways and thus promotes pathogenesis at various stages, form early infection to latency to granuloma formation. Here we discuss the present status of mycobacteria engaged Coronin 1-dependent signaling cascades and secreted PknG related sequence of events promoting mycobacterial pathogenesis. Current knowledge about these two proteins in context of macrophage signaling manipulation encompassing diverse mechanisms like calcium-calcineurin signaling, reduced proinflamtory cytokine secretion, cytoskeletal changes, and adaptation in acidic environment, which ultimately converge toward mycobacterial survival inside the macrophages has been discussed.

## Introduction

**Figure 1:**
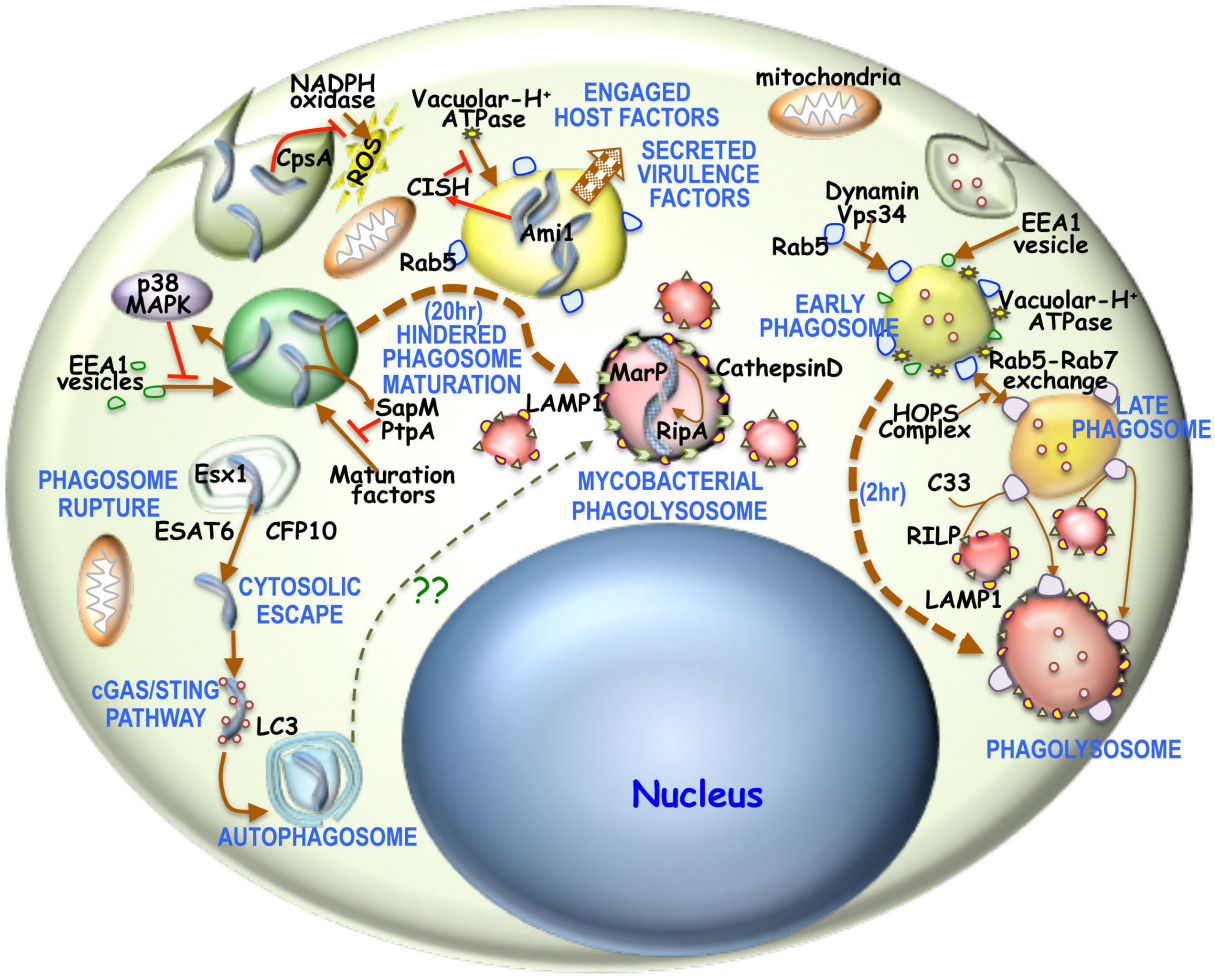
Overview of phagosome maturation in general or in mycobacterial infection: Generally upon phagocytic uptake, dynamin, and Vps34 enable fusion of Rab5 vesicles with the phagosome. There is concomitantly acquisition of EEA1 as well. This early phagosome is then acidified by V-H+ATPase, thus activating the HOPS complex to enable the Rab5-Rab7 exchange. The Rab7 positive acidified late endosome is then sensed by C33, which then enables the fusion of RILP positive lysosomes with these late endosomes to form the phagolysosome. It is believed that the process of phagosome to phagolysosome occur in 2–3 h. For mycobacterial phagosomes, NADPH oxidase activity is prevented by mycobacterial CpsA. Next mycobacteria activated p38-MAPK pathway hinders EEA1 acquisition onto these phagosomes. Concomitantly mycobacteria activated CISH can prevent phagosomal acidification by degrading V-H+ATPase. Mycobacteria secreted SapM and PtpA, limit the access of maturation factors thus hindering phagosome maturation. Part of the mycobacteria can escape into the cytosol by rupturing the maturation hindered phagosome through Esx1 secreted ESAT6 and CFP10. Cytosolic mycobacteria triggers the cGAS/STING pathway and thereby forms an LC3 positive autophagosome. Hindered phagosome exhibit phagolysosomal characteristics in being LAMP1 and CathepsinD positive 20 h post-phagocytosis of mycobacteria. This hindrance is required to overexpress acid tolerant protease MarP, which then activates RipA. Ami1 together with activated RipA then promotes mycobacterial replication and proliferation.

Macrophages are the first line of defense to any intracellular microbial attack in our body and to stifle these microbial invaders, macrophage have evolved different innate immune strategies like phagocytosis, micropinocytosis, phagosome-lysosome fusion, apoptosis, autophagosome formation, antigen presentation etc. (Weiss and Schaible, 2015; BoseDasgupta and Pieters, 2018). This panoply of innate immune events orchestrated by the macrophage, ensures microbial clearance form our body. However, macrophages in certain cases are incapable of eliminating the invaded pathogen, thus allowing them to form a safe niche within itself, which ultimately emanates into a diseased condition. *Mycobacteria sp*. which are the causative agent of pulmonary and extra-pulmonary tuberculosis, leprosy, skin abscess etc. are one of those notorious intracellular pathogens. On average 10 million people contract tuberculosis every year with a mortality being close to 1.5 million. Additionally latent TB infection globally is 1.7 billion of which more than 10% progress to active disease (World Health Organization, 2019). India harbors one-third of global TB burden of which 40% is infected with XDR/TDR mycobacterial strains. It is believed that more than 40% of Indian population are effected with latent TB (Goyal et al., 2017). On the otherhand there are close to 0.2 million leprosy cases and the count seems to rise slowly with approximately one in fifty thousand getting affected. *M. tuberculosis* is transmitted from an active TB patient through the aerosolized droplets released upon sneezing or coughing, which when inhaled by another person migrates through the respiratory track to the lower lobe of lungs where it recruits and infects alveolar macrophages. *M. leprae* is transmitted through nose and mouth droppings from infected individuals. When macrophage fail to prevent mycobacterial pathogenesis within itself, the immune system tries to contain mycobacteria by forming a fibroblast coat around the infected macrophages, thus forming a granuloma. Formation of granuloma indicates onset of active TB, which progresses as a necrotizing caseous core generated by macrophage rupture of mycobacteria followed by mycobacterial dissemination to other loci for disease progression (Cambier et al., 2014). This intelligent acid-fast bacilli have evolved certain strategies to withstand macrophage laid elimination mechanisms and in contrary exploit these cells as a protective shield by secreting certain virulence factors and hijacking macrophage proteins (Pieters, 2008). Upon mycobacterial apposition close to macrophage membrane, depending on the context, different phagocytic receptors such as Complement receptor 3, mannose receptor, Sp-A receptor, Fcg receptor, Dectin 2, Mincle, and TLR2 recognize mycobacteria (Caron and Hall, 1998; Killick et al., 2013; Hmama et al., 2015; Wagener et al., 2018) and trigger membrane invagination and pseudopod extension along the mycobacterial surface. In general upon phagocytic uptake or even before phagocytic cup closure, few proteins like NADPH oxidases (Panday et al., 2015) are recruited and activated, while expression of pro-inflammatory cytokines (Domingo-Gonzalez et al., 2016) occur so as to initiate an antimicrobial response. NADPH oxidase is known to generate ROS and thereby activate the innate immune system through a Th1 response. But mycobacteria is known to utilize CpsA to prevent the action of NADPH oxidase (Köster et al., 2017). *Mycobacteria* is known to engage host factors such as Coronin 1, Calcineurin, SOCS1, CISH, LRG47, Gbp5 etc. to establish its niche within the macrophage. Trimeric Coronin 1 being cortical gets associated with the phagsome and is retained by mycobacteria to hinder phagosome maturation through activation of the phosphatase Calcineurin. *Mycobacteria* by upregulating SOCS1 can prevent the downstream signaling of the pro-inflammatory cytokine IFNγ, whereas CISH has been shown to hinder the phagosomal localization of V-H+ATPase by degrading its subunit A and thereby hindering mycobacterial phagosome acidification and maturation (Jayachandran et al., 2007; Queval et al., 2017). Both LRG47 and Gbp5 are IFNγ inducible genes, where the later is triggered by mycobacteria secreted ESAT6 (MacMicking et al., 2003; Shenoy et al., 2012; Tretina et al., 2019). These proteins can activate the inflammasome pathway during mycobacterial infection (Tretina et al., 2019) while cytosolic mycobacteria can trigger autophagy (Gutierrez et al., 2004). Autophagy can dampen inflammasomes (Songane et al., 2012; Saitoh and Akira, 2016), thus creating a favorable niche for infected mycobacteria. *Mycobacteria* secretes a number of virulence factors such as PknG, PtpA, SapM, Eis etc, to promote pathogenesis. Where PknG is known to hinder phagolysosome fusion (Walburger et al., 2004), its role as a kinase inside macrophages is yet to be deciphered. PtpA on the other hand by dephopsphorylating VPS33b, excludes V-H+ATPases from acidifying the mycobacterial phogosome (Wong et al., 2011). Additionally, acid phosphatase SapM, by dephosphorylating the phosphoinositides can limit the phagosomal access of maturation factors and thereby hinder the process (Zulauf et al., 2018). Eis on the other hand alter the innate immune responses like autophagy, inflammation and cell death via a redox dependent manner (Shin et al., 2010). Following phagocytosis, dynamin, and Rab5 effector rabaptin engages the PI3 kinase; Vps34 to recruit Rab5 to the phagosomal membrane (Kinchen and Ravichandran, 2008). Concomitantly there is fusion of early endosomal antigen (EEA1) containing vesicles with these nascent phagosomes (Lawe et al., 2002). But for mycobacteria containing phagosomes, Vps34 generated PIP2 and PIP3 (Jeschke et al., 2015) is depleted by mycobacteria secreted SapM (Vergne et al., 2005) thus preventing the phagosomal access of maturation factors. Additionally mycobacteria activated p38-MAPK can reduce EEA1 recruitment to the mycobacterial phagosome (Fratti et al., 2003). In a poorly understood “early” acidification event, phagosomes are minimally acidified (Beyenbach and Wieczorek, 2006) which possibly triggers cathepsins to bring in V-H+ ATPase to the phagosome membrane (Fratti et al., 2003). Generally phagosomal acidification by V-H^+^ ATPase brings in the HOPS complex that interact with phagosome bound Rab5 and exchanges it with Rab7 to form the late phagosome (Caplan et al., 2000). These late phagosomes bring C33 protein that enables the interaction of lysosomal RILP protein with phagosome bound Rab7 and triggers phagolysosome formation (Jordens et al., 2001) which is marked by LAMP1 (Huynh et al., 2007). Owing to impaired acidification of mycobacteria containing phagosomes the HOPS complex might not be activated and therefore the Rab5 to Rab7 exchange and the process thereafter does not occur (Poirier and Av-Gay, 2012; Mottola, 2014). Although an insignificant level of Rab5 gains access to this phagosome it might be inefficient to recruit the HOPS complex to get exchanged with Rab7 (Mottola, 2014). Interestingly 20 h post-infection it has been observed that mycobacteria resides in LAMP1 and CathepsinD positive phagolysosomes (van der Wel et al., 2007). Hindered phagosome maturation (Ehrt and Schnappinger, 2009) might allow for the mycobacterial expression of MarP, a serine protease responsible for cleavage mediated activation of the peptidoglycan hydrolase RipA, that is essential for proper division of mycobacteria (Botella et al., 2017). RipA and Ami1 are essential for mycobacterial replication and persistence inside these phagolysosomes (Healy et al., 2020). Additionally a part of the infected mycobacteria is known to utilize the Esx1 secretion system to secrete ESAT6 and CFP10 to rupture the phagosome and escape into the cytoplasm (van der Wel et al., 2007). Once in the cytoplasm, these mycobacteria trigger the cGAS/STING pathway for autophagosome formation (Watson et al., 2015). The above stated observations are represented schematically in Figure 1. Once phagocytosed, infected mycobacteria face a hypoxic challenge, while withstanding a strong innate immune response or when it is restricted within the granuloma (Rustad et al., 2009). This hypoxic challenge triggers the DosR regulon, which then in a concerted way limit the mycobacterial metabolism and thereby its proliferation and trigger a condition of latency (Doddam et al., 2017). Mycobacteria infected macrophages, during the process of hindered phagosome maturation or cytosolic escape or upon induction of latency do not undergo apoptosis, while in general when macrophage fail in its ability to limit a pathogen it undergoes apoptosis. Mycobacteria are known to engage NuoG, SecA2, PknE, and SodA like proteins to limit macrophage apoptosis (Liu et al., 2017). Prevention of apoptosis restricts macrophage bactericidal activity and also allows mycobacteria to proliferate in its established niche in case of active TB or stay dormant as in the case of latent TB.

Thus, mycobacteria need to manipulate macrophage signaling cascades in a balanced way allowing it to get associated with the macrophage. Once inside the phagosome of permissive macrophages, mycobacteria recruits and retains the cortical protein, Coronin 1 on the phagosome membrane and secretes virulence factors of which Protein Kinase G (PknG) plays a primary role and these together enables it to survive within the macrophage as well as generate an anti-inflammatory milieu. Here we discuss different approaches taken by several groups to understand the role of Coronin 1 and PknG in mycobacterial pathogenesis.

## Coronin 1: the “Fifth Columnist” Inside Macrophages

Mycobacterial pathogenesis depends upon successful survival and replication of bacilli inside macrophages (Falkow, 1991). To fulfill this purpose *Mycobacterium sp* hijacks several host factors. Coronin 1 is one such factor that helps mycobacteria to prevent phagosome maturation and thereby aids in mycobacterial survival inside the macrophages (Hasan et al., 1997; Ferrari et al., 1999). Coronin 1 is an evolutionary conserved protein belonging to the β-propeller and WD repeat containing Coronin family protein (De Hostos, 1999). Coronin 1 is specifically expressed in the hematopoetic cell line lineage and to a lesser extent in the brain (Appleton et al., 2006; Jayachandran et al., 2014). Deletion of Coronin 1 affects F-actin dynamics and thereby compromises phagocytosis (Yan et al., 2005). Intracellularly, Coronin 1 trimerizes via its C-terminal coiled-coil domain and localizes to the cell cortex (BoseDasgupta and Pieters, 2014a), hence macrophage phagocytosis of live or dead mycobacteria has Coronin 1 recruited on the phagosome membrane, but only live pathogenic mycobacteria can retain this Coronin 1 coat (Ferrari et al., 1999). Live pathogenic mycobacteria secretes lipoamide dehydrogenase or CIP50, which then interacts with Coronin 1 across the phagosome membrane and thereby retains it on the phagosome (Deghmane et al., 2007). Interestingly pro-inflammatory cytokine IFNγ induced GTPase, LRG47 or called Immunity Related GTPase M (IRGM) in mice, can disrupt the interaction between lipoamide dehydrogenase and Coronin 1. The resultant loss of Coronin 1 from the phagosomal membrane enables maturation of the mycobacteria-containing phagosome and later fusion with the lysosomes where they are subsequently degraded by lysosomal hydrolases (Deghmane et al., 2007). Active TB patients exhibit elevated levels of Coronin 1 and TLR2 and it is believed that the former can trigger the overexpression of the later (Constantoulakis et al., 2010). *M. leprae* containg phagosomes have been shown to harbor both TLR2 and Coronin 1 on the phagosome membrane where the former trigger the innate immune response (Suzuki et al., 2006).

**Figure 2:**
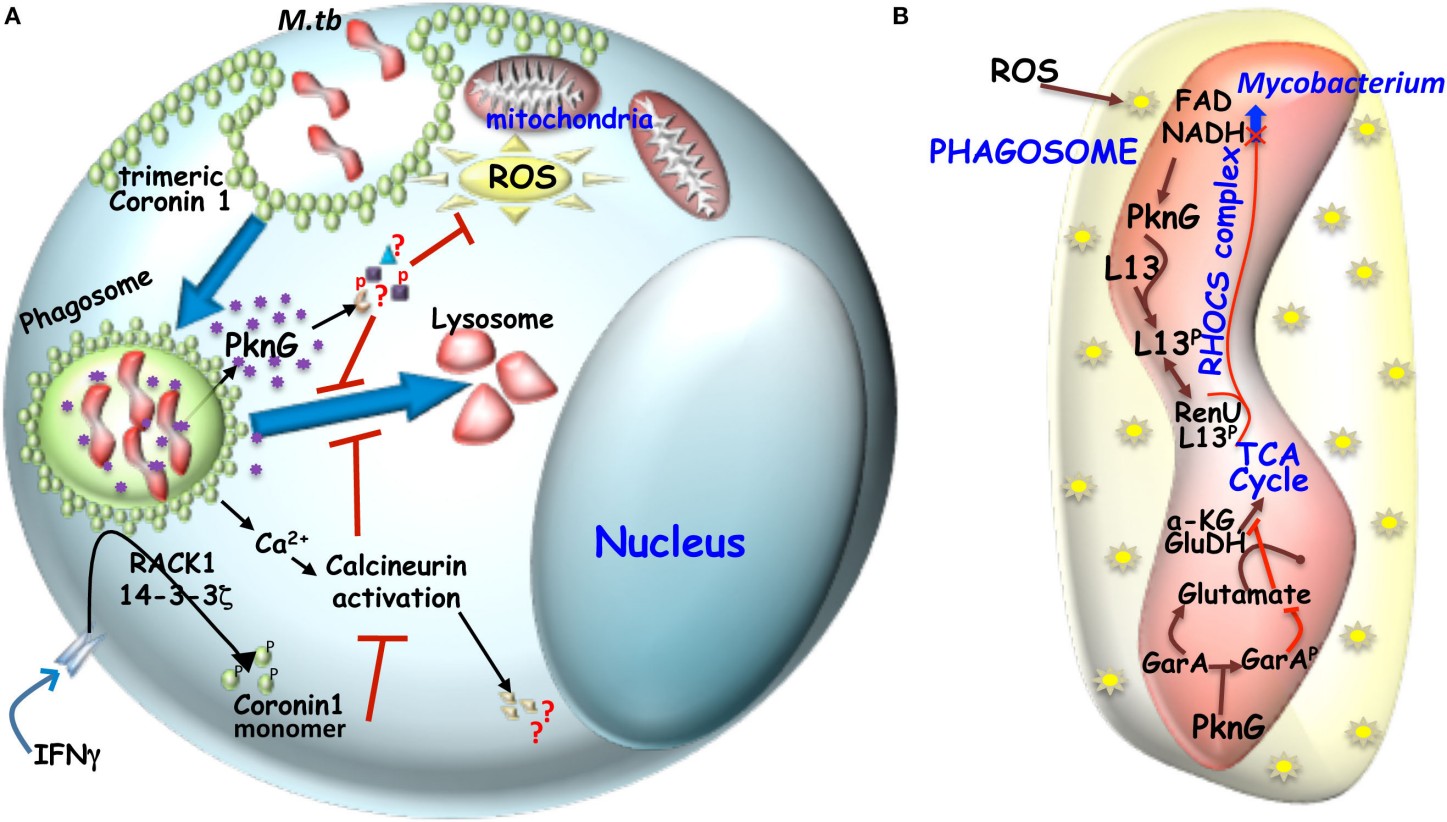
**(A)** Mechanism of hindered phagosome maturation: Signaling molecules like trimeric Coronin 1 (macrophage factor) and mycobacteria secreted virulent kinase, PknG hinders phagosome maturation. Trimeric coronin1 activates the phosphatase calcineurin by secreting calcium and thus maintains the trimeric scaffold of Coronin 1 around the phagosome while PknG exerts its effect by phosphorylating unknown effector molecules inside the macrophage. **(B)** Metabolic adaptation of mycobacteria to intracellular ROS: Mycobacterial infection induced ROS can increase NADH level inside mycobacteria, which then engages the RHOCS comlpex. Increased NADH activated PknG phosphorylates L13a, which then interacts with RenU and the complex then degrades NADH. In parallel activated PknG also phosphorylates GarA. While unphosphorylated GarA can hinder the TCA cycle by engaging the enzymes α-KG and glutamate dehydrogenase (GH) to produce glutamate and aspartate, but when phosphoryated α-KG and GH are free to act in TCA cycle and the glutamate and aspartate levels drop.

Kuffer cell or liver macrophages, which interestingly do not express Coronin 1, can efficiently eliminate phagocytosed mycobacteria through lysosomal transfer (Anand and Kaul, 2005). Trimeric Coronin 1 is known to induce phagosome maturation arrest via activation of calcium dependent phosphatase calcineurin (Jayachandran et al., 2007). Coronin 1 regulates intracellular Ca^2+^ homeostasis by promoting Ca^2+^ release through CRAC channels (Pieters et al., 2013) thus forming local Ca^2+^ pool around the phagosome that could be activating calcineurin, which then by dephosphorylating a group of proteins called “dephosphins” could hinder phagosome maturation. Hence Calcineurin inhibitor FK506 pre-treated macrophages upon being infected with mycobacteria, results in phagosome maturation (Jayachandran et al., 2007). Phagosomal Coronin 1 is known to block p38-MAPK triggered LC3 recruitment to the phagosomes and autophagosome formation (Seto et al., 2012), which is crucial to mycobacterial pathogenesis. Interestingly the C-terminal coiled-coil deleted Coronin 1 expressing Coronin 1 knockdown cells or pro-inflammatory cytokine activated macrophages upon mycobacterial infection fail to activate Calcineurin, thus indicating the essentiality of the trimeric form of Coronin 1 for activation of Calcineurin (BoseDasgupta and Pieters, 2014b). Pro-inflammatory cytokine activated macrophages induces Coronin 1 phosphorylation by PKC on serine residues in the C-terminal coiled-coil domain (BoseDasgupta et al., 2015) to render it monomeric probably due to the steric repulsion of positively charged coiled-coil domain through which trimerization occurs. Monomeric Coronin 1 being unstable is scaffolded by RACK1 and this complex is shuttled from the cortex to the cytoplasm by the cargo carrier 14-3-3ζ (BoseDasgupta et al., 2015). Hence for classically activated macrophages pathogenic uptake switches from phagocytosis to macropinocytosis wherein the mycobacteria containing macropinosomes lack the trimeric Coronin 1 scaffold, the ensuing calcineurin activation and thereby gets transferred to lysosomes and eliminated (BoseDasgupta and Pieters, 2014b). The above mentioned mechanisms are represented schematically in Figure 2A.

Further it has been shown that compounds that downregulate coronin 1 has the potential to inhibit mycobacterial survival inside macrophages (Anand et al., 2006; Kaul, 2008). Vitamin D3 and retinoic acid, activates VDR and RXR, which exhibits a synergistic action by forming the heterodimer VDR/RXR that competes with NFAT1/AP1 to bind to its sequence and thereby repress Coronin 1 expression (Anand and Kaul, 2003). It has been observed that mycobacterial infection reduces intracellular VDR levels, and thereby maintain Coronin 1 levels in the cell (Salamon et al., 2014). *M. tuberculosis* also exploits Coronin 1 as a defense mechanism against autophagy (Seto et al., 2012). Autophagosome formation through recruitment of several layers of membrane around the phagosome is prevented by mycobacteria containing phagosomes harboring the Coronin 1 coat and downregulation of Coronin 1 resulted in LC3 localization to these mycobacteria containing phagosomes and further maturation and fusion with the lysososme resulting in mycobacterial elimination (Seto et al., 2012). Interestingly mycobacteria that escape into the cytosol trigger autophagic response through recruitment of p62/TBK1 like proteins and result in the transfer of these mycobacteria to lysosomes (Pilli et al., 2012). Activation of p38 MAPK is essential for autophagosome formation. Coronin 1 could be blocking the activation of p38 MAPK pathway, which occurs through its phosphorylation, as Coronin 1 knockdown macrophages exhibit increased levels of phosphorylated p38 MAPK (Seto et al., 2012). Taken together it is evident that cortical trimeric Coronin 1 is an immunologically essential host protein is efficiently exploited by mycobacteria to establish its niche within the macrophage.

## Mycobacteria Secreted Pkng, a Versatile Weapon Against Macrophage Signal Modulation

The genome of mycobacteria harbors 11 eukaryote-like serine/threonine kinases of which, Protein kinase G (82 kda) is one (Cole et al., 1998). The PknG gene locus although being conserved throughout the mycobacterial genus, is believed to be expressed predominantly in the pathogenic forms. Lysine181 in ATP-binding pocket of PknG acts as the active site residue whose mutation renders it inactive (Koul et al., 2001). Mycobacteria secreted PknG shares structural similarity to *Yersinia* secreted pathogenesis associated kinase YOPO thus indicating the role of PknG in mycobacterial pathogenesis (Aslund and Beckwith, 1999). Presence of PknG gene in the glutamate binding protein operon of H37Rv suggests that it is associated with virulence through regulation of glutamate metabolism (Bhattacharyya et al., 2018). Simultaneously PknG was shown to be secreted inside macrophages to prevent phagosome-lysosome fusion (Cowley et al., 2004; Walburger et al., 2004). Compared to wild type, PknG deleted pathogenic mycobacteria gets rapidly transferred to lysosomes and degraded (Walburger et al., 2004). During the course of evolution, mycobacteria assimilated several host-kinases through horizontal and lateral gene transfer and evolved them to manipulate host cell signaling and vesicular trafficking pathways. Structurally PknG has an unique multidomain make-up, starting with an extremely unstable N-terminus, followed by the autophosphorylation domain, thereafter the rubridoxin domain, next a central kinase domain, thereafter a tetratricopeptide repeat (TPR) domain and finally the C terminus (Scherr et al., 2007; Lisa et al., 2015). TPR domain, a consensus repeat sequence of 34 degenerate amino acids are generally involved in protein-protein interaction (Scheufler et al., 2000). Therefore, PknG could be interacting with other proteins via its TPR domain to manipulate host cell signaling proteins. The kinase domain of PknG has been shown to interact with macrophage Rab7la-GDP in the trans Golgi network, causing inhibition of GTP-GDP exchange and thereby impairing Rab-GTP recruitment to the lysosomal membrane and thus preventing phagosome-lysosome fusion. This interaction also impairs the recruitment of EEA1 and other proteins crucial for phagosome maturation (Pradhan et al., 2018). As a kinase the main function of PknG should be to phosphorylate macrophage proteins, since PknG does not phosphorylate Rab7la it could be postulated that this interaction driven role in hindering phagosome maturation could be a bystander function of PknG and its major role inside macrophages is yet to be deciphered.

PknG gets transphosphorylated on N-terminal threonine residues prior to activation of the kinase domain for phosphorylation events and thereby accentuate mycobacterial survival inside macrophages (Scherr et al., 2009). This transphosphorylation, aided by the rubridoxin domain, is thought to properly structure the N-terminal disordered region and thereby help PknG attain a substrate binding conformation and enable its phosphorylation (Tiwari et al., 2009; Wittwer et al., 2016). The rubridoxin domain harbors four cysteine residues, which forms an iron-sulfur cluster (Wittwer et al., 2016). Such clusters regulate protein conformation which is triggered by the S-nitrosylation of one or more of the cysteine residues in the cluster (Saini et al., 2012). Mutation in these cysteine residues of PknG makes it insensitive to surrounding redox environment (Wittwer et al., 2016). Mycobacterial infection of macrophages causes an intracellular stress, which then leads to production of redox intermediates in the form of ROS and RNI as a major defense mechanism (Kumar et al., 2011). To combat this ROS and RNI mediated stress, mycobacteria employs redox sensors called RHOCS (Wolff et al., 2015) consisting of PknG, ribosomal protein L13 and RenU (nudix hydrolase), that enable metabolic adaptation (Wolff et al., 2015). Macrophage ROS induced increased NADH levels inside the mycobacteria upregulates PknG, which then phosphorylates L13, to induce its association with RenU and the complex then brings NADH to its normal level. Deletion of PknG causes impairment of RHOCS leading to increase susceptibility of mycobacteria to oxidative stress (Wolff et al., 2015). PknG contributes to intramacrophage metabolic adaptation via GarA phosphorlytion. Unphosphorylated GarA upregulates glutamate synthesis and inhibits TCA cycle by directly binding to alpha-ketoglutarate and glutamate dehydrogenase complex, while phosphorylated GarA stimulates aspartate and glutamate catabolism causing a shift of metabolism required for efficient growth of mycoabcteria inside the macrophages and for successful pathogenesis in mice (Rieck et al., 2017). The role of PknG inside mycobacteria has been depicted schematically in Figure 2B. Supply of amino acid abrogates the effect of GarA deletion, thus suggesting that mycobacteria can efficiently sense amino acid availability inside macrophages and thereby adapting it to intercellular lifestyle. Deletion of PknG confines macrophage derived amino acid utilization and encourages lower metabolic activity with respect to PknG expressing wild type mycobacteria thus promoting latency-like conditions inside macrophages (Khan et al., 2017; Rieck et al., 2017). Only pathogenic mycobacteria can downregulate PKCalpha expression inside macrophages due to secretion of PknG inside these macrophages (Chaurasiya and Srivastava, 2009). Protein microarray analysis recently identified Cyclophilin A, a macrophage cytoplasm abundant protein to be interacting with PknG both *in vitro* and upon infection with mycobacteria (Wu et al., 2018). Cyclophilin A (CypA) which is secreted in response to inflammatory stimuli gets degraded upon being phosphorylated by PknG and this inhibits the inflammatory response through suppression of NF-κB and ERK1/2 pathway. In macrophages PknG overexpression decreases intracellular cytokine levels, thus promoting mycobacterial survival (Wu et al., 2018). Since PknG is an important secreted virulence factor crucial for mycobacterial pathogensis several groups have screened for inhibitors against it. Tetrahydrobenzothiophene (AX20017) was identified as a specific and potent, ATP competitive inhibitor of PknG kinase activity, capable of inducing phagosome maturation (Scherr et al., 2007). Recently, Sclerotiorin a marine seaweed derived compound (Chen et al., 2017) and NU-6027 (4-cyclohexyl, 2-6-diamino-5-nitrosopyrimidine) (Kidwai et al., 2019) a known CDK-2 inhibitor were also found to inhibit the autophosphorylation of PknG, which then leads to lysosomal transfer of infected mycobacteria. Altogether it is established that mycobacteria secreted PknG plays a crucial role in mycobacterial pathogenesis and thus an important therapeutic target in a realm of drug resistance to curb the global tuberculosis menace.

## Conclusion

The interplay of host factors and secreted virulence factors culminating in immunesubversion ultimately enables successful mycobacterial pathogenesis. Owing to the emergence of chemoresistant mycobacterial species and the success of host-directed immunomodulatory therapeutics in oncotherapy, such approaches are being envisioned against tuberculosis and leprosy. Targeting of Coronin 1 trimerization through peptidomimetics could induce maturation of mycobacterial phagosomes thus leading to lysosomal transfer and elimination. PknG being a secreted kinase can be targeted in the macrophage cytosol so as to prevent phosphorylation of host substrates key to mycobacterial pathogenesis and thereby induce its elimination. Therefore, host-directed therapeutics would emanate as an efficient strategy to combat the global tuberculosis and leprosy menace.

## Author Contributions

SS wrote the manuscript. SB reviewed the manuscript and provided critical inputs. PD checked for format and typographical errors. All authors contributed to the article and approved the submitted version.

## Conflict of Interest

The authors declare that this mini review was prepared in the absence of any commercial or financial relationships that could be interpreted as a potential conflict of interest.
